# Cutaneous nociception evoked by 15-delta PGJ2 via activation of ion channel TRPA1

**DOI:** 10.1186/1744-8069-4-30

**Published:** 2008-07-31

**Authors:** Lillian Cruz-Orengo, Ajay Dhaka, Robert J Heuermann, Timothy J Young, Michael C Montana, Eric J Cavanaugh, Donghee Kim, Gina M Story

**Affiliations:** 1 Washington University Pain Center, Department of Anesthesiology, Washington University School of Medicine, St. Louis, MO, 63130, USA; 2 Department of Cell Biology, The Scripps Research Institute, La Jolla, CA, 92037, USA; 3 Department of Physiology, Rosalind Franklin University of Medicine and Science The Chicago Medical School, North Chicago, IL, 60064, USA

## Abstract

**Background:**

A number of prostaglandins (PGs) sensitize dorsal root ganglion (DRG) neurons and contribute to inflammatory hyperalgesia by signaling through specific G protein-coupled receptors (GPCRs). One mechanism whereby PGs sensitize these neurons is through modulation of "thermoTRPs," a subset of ion channels activated by temperature belonging to the Transient Receptor Potential ion channel superfamily. Acrid, electrophilic chemicals including cinnamaldehyde (CA) and allyl isothiocyanate (AITC), derivatives of cinnamon and mustard oil respectively, activate thermoTRP member TRPA1 via direct modification of channel cysteine residues.

**Results:**

Our search for endogenous chemical activators utilizing a bioactive lipid library screen identified a cyclopentane PGD_2 _metabolite, 15-deoxy-Δ^12,14^-prostaglandin J_2 _(15d-PGJ_2_), as a TRPA1 agonist. Similar to CA and AITC, this electrophilic molecule is known to modify cysteines of cellular target proteins. Electophysiological recordings verified that 15d-PGJ_2 _specifically activates TRPA1 and not TRPV1 or TRPM8 (thermoTRPs also enriched in DRG). Accordingly, we identified a population of mouse DRG neurons responsive to 15d-PGJ_2 _and AITC that is absent in cultures derived from TRPA1 knockout mice. The irritant molecules that activate TRPA1 evoke nociceptive responses. However, 15d-PGJ_2 _has not been correlated with painful sensations; rather, it is considered to mediate anti-inflammatory processes via binding to the nuclear peroxisome proliferator-activated receptor gamma (PPARγ). Our *in vivo *studies revealed that 15d-PGJ_2 _induced acute nociceptive responses when administered cutaneously. Moreover, mice deficient in the TRPA1 channel failed to exhibit such behaviors.

**Conclusion:**

In conclusion, we show that 15d-PGJ_2 _induces acute nociception when administered cutaneously and does so via a TRPA1-specific mechanism.

## Background

The prostaglandins (PGs) are a class of biomolecules derived from arachidonic acid (AA) that are involved in a variety of signaling processes including inflammation. For example, PGE_2 _and PGI_2 _are produced during inflammation and contribute to the direct sensitization of nociceptive neurons of the dorsal root ganglia (DRG). Downstream of binding to its G protein-coupled receptor (GPCR), PGE_2 _sensitizes nociceptive neurons to thermal stimuli via PKA-dependent phosphorylation of the heat- and capsaicin-gated Transient Receptor Potential (TRP) ion channel TRPV1 [1]. TRPV1 is the founding mammalian member of a subfamily of TRP channels gated by temperature (dubbed thermoTRPs)[2].

TRPA1, first characterized as a thermoTRP channel gated by noxious cold (although this finding is controversial) [3] is activated by compounds that induce "burning" sensations and therefore could be best classified as a "chemoTRP." Irritant chemicals ligands of TRPA1 include allyl isothiocyanate (AITC), cinnamaldehyde (CA), allicin, acrolein and formalin[4-8]. TRPA1 is also tightly coupled to bradykinin signaling and is activated by agents generated by oxidative stress[4,7,8]. Two groups have recently shown that covalent modification of cytoplasmic N-terminal cysteine residues via the Michael addition reaction is a common mode of action of several TRPA1 agonists[9,10].

In order to identify novel and endogenous TRPA1 activators, we performed a bioactive lipid library screen and describe here our findings that 15-deoxy-Δ12,14-prostaglandin J_2 _(15d-PGJ_2_) a cyclopentane prostaglandin metabolite of PGD_2_[11], specifically activates this channel and not other thermoTRPs enriched in DRG. Similar to AITC and CA, 15d-PGJ_2 _is characterized by an electrophilic α,β-unsaturated carbonyl group capable of undergoing a Michael addition with nucleophilic groups on cysteine residues. A role of 15d-PGJ_2 _in peripheral nociception has not been described previously; rather this molecule is best characterized as an anti-inflammatory agent[12]. However, we have identified a population of DRG nociceptive neurons that respond to 15d-PGJ_2 _and the TRPA1-specific agonist AITC. We therefore further hypothesized that 15d-PGJ_2 _plays an *in vivo *role in acute peripheral nociceptive signaling via TRPA1 activation. Accordingly, we found that 15d-PGJ_2 _induced acute nociceptive responses when administered cutaneously in mice. This effect is specific to TRPA1 as these nociceptive behaviors are significantly attenuated in TRPA1 knockout mice. Taken together our results demonstrate a novel TRPA1-dependent role of 15d-PGJ_2 _in acute pro-nociception.

## Materials and methods

### Bioactive Lipid Library Screen

Intracellular calcium measurements were performed using a Fluorometric Imaging Plate Reader (FLIPR). Mus musculus TRPA1 (mTRPA1)-CHO cells were seeded at 6000 cells/well into black-walled base 384-well plates and were grown for 2 days. Cells were induced for mTRPA1 expression as described and loaded with Fluo-3 according to protocol (Molecular Probes). The plates were placed into a FLIPR (Molecular Devices, UK) to monitor cell fluorescence (EX _ 488 nM; EM _ 540 nM) before and after the 201 lipids contained in the Biomol Bioactive Lipid Library were added[4].

### Electrophysiology

HeLa cells were seeded at a density of 2 × 10^5 ^cells per 35 mm dish 24 hr prior to transfection in Dulbecco's modified Eagle's medium (DMEM) containing 10% fetal bovine serum. Cells were co-transfected with plasmids containing mTRPA1, mTRPV1 or mTRPM8 and GFP (green fluorescent protein) in pcDNA3.1 using LipofectAMINE and OPTI-MEM I Reduced Serum Medium (Life Technologies). Green fluorescence from cells expressing GFP was detected with the aid of a Nikon microscope equipped with a mercury lamp light source and a GFP filter (emission wavelength, 510 nm). Cells were used 1–2 days after transfection. Only cells showing normal ellipsoidal shape were used.

Gigaseal was formed with pipettes with desired resistance (2–5 Mohms). Current was recorded with an Axopatch 200 patch-clamp amplifier, low-pass filtered at 3 KHz using an 8-pole Bessel filter (902-LPF), digitized using Digidata1322A, and stored on computer disk. Digitized data were analyzed (pClamp 9.0) to obtain channel activity (NP_o_; where N is the number of channels in the patch and P_o _is the open probability), and amplitude histograms to obtain single channel conductance. Current tracings shown in figures have been filtered at 1 KHz. For whole-cell recordings, bath solution contained 126 mM NaCl, 4 mM KCl, 2 mM EGTA, 1 mM MgCl_2_, 10 mM HEPES, 5 mM glucose, and pipette solution contained 130 mM CsCl, 2 mM EGTA, 1 mM MgCl_2_, 2 mM ATP and 100 mM GTP and 10 mM HEPES (pH 7.3). For cell-attached patches, pipette and bath solutions contained (in mM): 126 mM NaCl, 4 mM KCl, 2 mM EGTA, 1 mM MgCl_2_, 10 mM HEPES, and 5 mM glucose (pH 7.3). Student's t test was used to test for significance (p < 0.05). All experiments were done at room and bath temperatures of 23 ± 1°C.

### DRG culture and intracellular calcium imaging

Calcium imaging experiments of DRG neurons were performed as described[4]. Briefly, DRG neurons from all spinal levels were rapidly dissected from adult mice and cultured for 24 h before assays were performed. All assays were performed in quadruplicate. For DRGs, 100 μM15d-PGJ2 (3 min pulse), 100 μM AITC (2 min pulse) and 1 μM capsaicin (2 min) were applied with a 4-min washout in between each stimulus.

### Behavioral Assays

Male C57BL6/J and TRPA1 mutant strain mice (obtained from David P. Corey and backcrossed to C57BL6/J for 5 generations) of 10–12 weeks age were used. Experimenters were blind with respect to genotype. Responses were averaged and analyzed using Student's t-test.

Animals were placed in individual Plexiglas boxes on a grid platform and habituated to the testing environment for one hour. After the habituation period, each mouse was injected on the plantar surface of the right hindpaw with 10 μl of 15 nmol 15d-PGJ_2 _diluted in 10% DMSO/normal saline (vehicle). This concentration was based on dose-response behaviors with injections ranging from 2.5–25 nmol concentrations of 15d-PGJ2 compared to vehicle. Behavior was recorded for 10 min after intraplantar injection. Observed variables were latency of response to licking and lifting of the paw as well as time spent licking/lifting of the paw.

### Compounds

AITC, menthol and capsaicin were purchased from Sigma Chemical Co. PGD2, PGJ2, delta12-PGJ2 and15d-PGJ2 were purchased from Biomol. For calcium imaging and electrophysiology experiments, PGs were dissolved in DMSO at a stock concentration of 20 mM and used at the final DMSO concentration of 0.1% or less.

## Results

### TRPA1 is specifically activated by 15d-PGJ2

**Figure 1:**
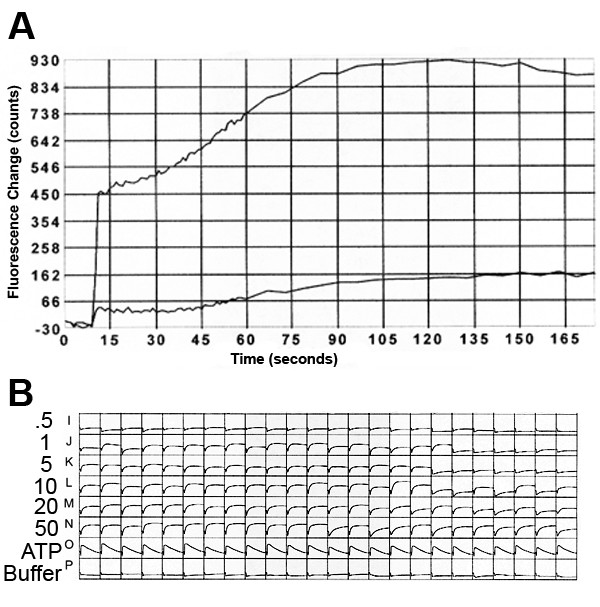
**FLIPR-based library screen of 15d-PGJ2 activation of TRPA1**. A. Fluorescence increases of TRPA1-expressing or mock transfected controls in response to a 50 μM 15d-PGJ2 stimulus. Traces represent average increases of cells from a 96-well plate. B. Fluorescence increases shown as minigraphs within 48 wells of TRPA1-expressing cells in response to increasing concentrations of 15d-PGJ2. All concentrations are in μM. 100 μM ATP and buffer were delivered as controls (the strain of CHO cell used exhibits endogenous ATP response and was used to ensure cell viability after compound addition).

**Figure 2:**
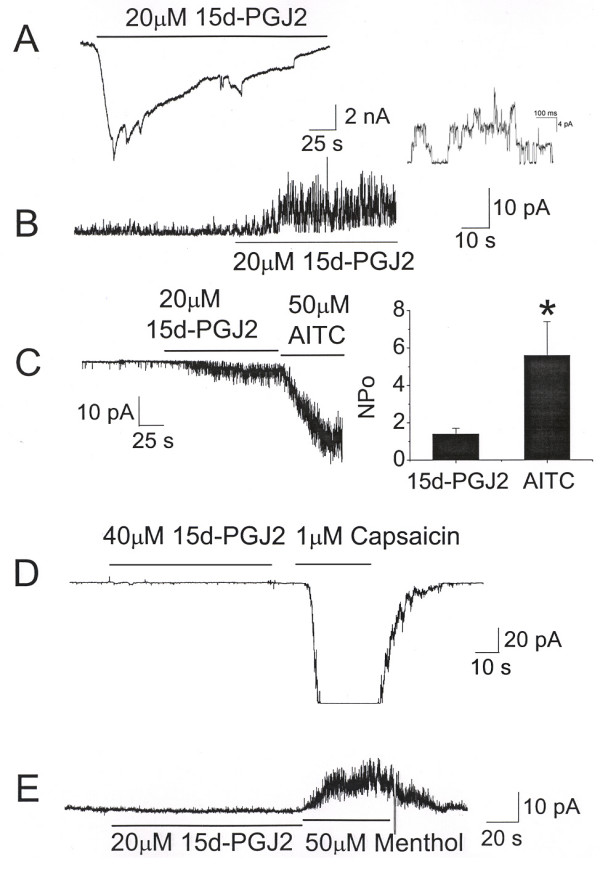
**15d-PGJ_2 _activates TRPA1, but not TRPV1 or TRPM8, expressed in HeLa cells**. A. Whole-cell current was recorded at a membrane potential of -40 mV (n = 3). B. A cell-attached patch shows activation of TRPA1 by 15d-PGJ_2_. Pipette potential was held at -60 mV to record outward current (n = 4). 15d-PGJ_2 _increased the channel activity (NPo) from a basal level of 0.03 ± 0.01 to 2.10 ± 0.30. C. A cell-attached patch shows activation of TRPA1 by 15d-PGJ_2 _and AITC. Pipette potential was held at +40 mV to record inward current. The bars in the graph represent the mean ± SD of 4 determinations, and are significantly different from each other. The channel activity (NPo) elicited by AITC was 6.6 ± 1.4. D-E. In HeLa cells expressing TRPV1 or TRPM8, 15d-PGJ_2 _had no effect, whereas capsaicin or menthol respectively, activated TRPV1 or TRPM8 (n = 4). Pipette potential was held at -40 mV. The large activation for TRPV1 by capsaicin could not be recorded at the amplifier gain setting used to show the lack of effect of 15d-PGJ2, but full washout was always observed following removal of capsaicin.

In order to identify novel TRPA1 agonists, we employed a Fluo-3 FLIPR-based screen of >200 bioactive lipids. This search identified that 15d-PGJ_2 _activated mTRPA1 in a dose dependent manner (Figure 1A–B). In these preliminary screen experiments, ATP was used as a control for cell viability as CHO cells show endogenous and robust responses to this compound. To determine whether 15d-PGJ_2 _directly activates TRPA1, whole-cell current was recorded from HeLa cells expressing mTRPA1 at a holding membrane potential of -40 mV. Application of 15d-PGJ_2 _to the bath solution rapidly increased the inward current that slowly decreased with time (Figure 2A). In the cell-attached patches used in this study, the number of TRPA1 channels expressed ranged from 3 to 14. In cell-attached patches with pipette potential held at -60 mV to record outward current, application of 15d-PGJ_2 _to the external solution also activated single channel currents in all patches tested (Figure 2B). Control solution containing 0.1% DMSO did not activate any channels (n = 8). Single channels activated by 15d-PGJ2 are shown at expanded time scale in the inset of Figure 2B. The single channel conductance of the inward current (+40 mV pipette potential) was 87 ± 3 pS (n = 3), based on measurements at two membrane potentials 0 and +40 mV. This is similar to the single channel conductance values reported earlier [13,14]. The single channel properties of TRPA1 expressed in HeLa cells have recently been characterized in detail [14] and therefore not described here. Because TRPA1 switches to a conformation that is insensitive to thiol-reactive compounds when inside-out patch is formed, all experiments were done using cell-attached patches. In cell-attached patches with pipette potential at +40 mV to record inward current, 15d-PGJ_2 _also activated TRPA1. Further addition of 50 μM AITC produced a much greater activation of TRPA1 (Figure 2C). In HeLa cells transfected with plasmid containing only GFP DNA, 15d-PGJ2 did not activate any channels (n = 5). As 50 μM AITC maximally activates TRPA1, these results show that the current activated by 20 μM 15d-PGJ_2 _is ~26% of the peak current (Figure 2C, inset) under these experimental conditions. As 15d-PGJ2 and AITC were added to the bath solution outside of the pipette, the result also shows that the compounds must cross the membrane and activate TRPA1 from the intracellular side of the membrane. The true concentration of PGJ2 in the cell that interacts with TRPA1 is difficult to know. Because the activation of TRPA1 by AITC was not easily reversible even after several minutes of washout, whether PGJ2 sensitized the action of AITC could not be determined in the same cell. In three cell-attached patches with 10 μM ruthenium red in the pipette, addition of 15d-PGJ2 failed to activate TRPA1 (data not shown), further showing that TRPA1 is the target of this prostaglandin. In cells expressing TRPV1, application of 15d-PGJ_2 _failed to activate TRPV1, but subsequent addition of capsaicin strongly activated the channels, as predicted (Figure 2D). Similarly, in cells expressing TRPM8, application of 15d-PGJ_2 _failed to activate any channels, but subsequent addition of menthol elicited strong activation (Figure 2E). Although other TRP ion channels were not tested, these results suggest that 15d-PGJ_2 _is specific for TRPA1 among three TRP channels tested.

**Figure 3:**
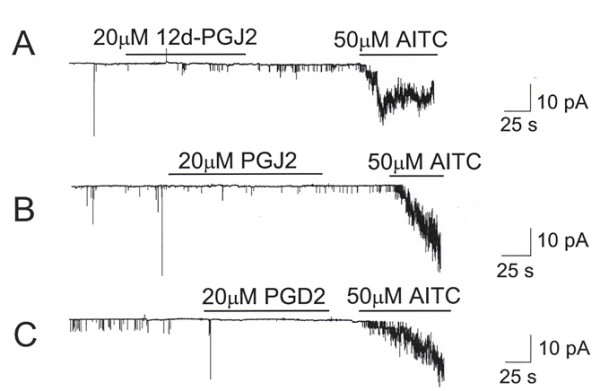
**PGD_2 _and its metabolites do not activate TRPA1**. A-C. Cell-attached patches show lack of activation of TRPA1 by 12d-PGJ_2_, PGJ_2 _and PGD2. In the same cells, AITC caused strong activation (n = 5 each). Pipette potential was held at +40 mV.

To test whether PGD_2 _itself or related PGD_2 _derivatives also activate TRPA1, we tested 12d-PGJ_2_, PGJ_2 _and PGD_2_. Similar to results obtained in our FLIPR-based library screen, none of these compounds were able to activate TRPA1 at similar concentrations, although AITC showed clear activation of TRPA1 in the same patches (Figure 3A–C). Under our experimental condition, it takes many minutes (~10 min) to washout the effect of AITC on TRPA1 and the recovery phase is not shown. Taken together, these data demonstrate that 15d-PGJ_2 _is a specific activator of TRPA1.

### TRPA1 is required for 15d-PGJ2 sensitivity of cultured DRG neurons

**Figure 4:**
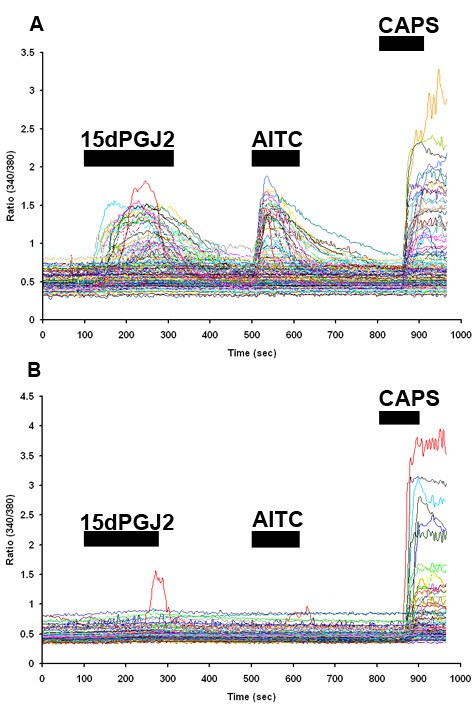
**15d-PGJ_2 _specifically activates TRPA1-expressing DRG neurons**. Traces represent increased fluorescence ratios of DRG neurons cultured from TRPA1 WT (A) or knockout (B) mice in response to 15d-PGJ_2_, AITC and capsaicin. Panels A and B illustrate responses of neurons from separate calcium experiments (~100 neurons per experiment are illustrated). All experiments were performed in quadruplicate from cultures derived from 10 animals per genotype. Compound application is indicated by black bars (Caps, Capsaicin).

To discover if 15d-PGJ_2 _activates TRPA1-expressing sensory neurons, we performed calcium imaging of cultured adult TRPA1 knockout and wildtype littermate DRG neurons in response to 15d-PGJ_2_, AITC and capsaicin. In support of specific activation of TRPA1, 100 μM 15d-PGJ_2 _induced increased fluorescence ratios (scored as 50% above baseline fluorescence) in 27% of cultured DRG neurons derived from wildtype mice (387 total neurons). Of these, 90% and 83% were AITC- and capsaicin-responsive respectively (Figure 4A). In DRG cultures (360 total neurons) derived from TRPA1 knockout mice, 100 μM 15d-PGJ_2 _and 100 μM AITC overall failed (2.3% and 0.5% of total, respectively) to elicit significant fluorescence increases, while capsaicin responses were intact (Figure 4B). These data show that 15d-PGJ_2 _specifically activates a subset of predicted TRPA1-expressing DRG neurons.

### 15d-PGJ2 elicits acute nociceptive behavior via a TRPA1-dependent mechanism

**Figure 5:**
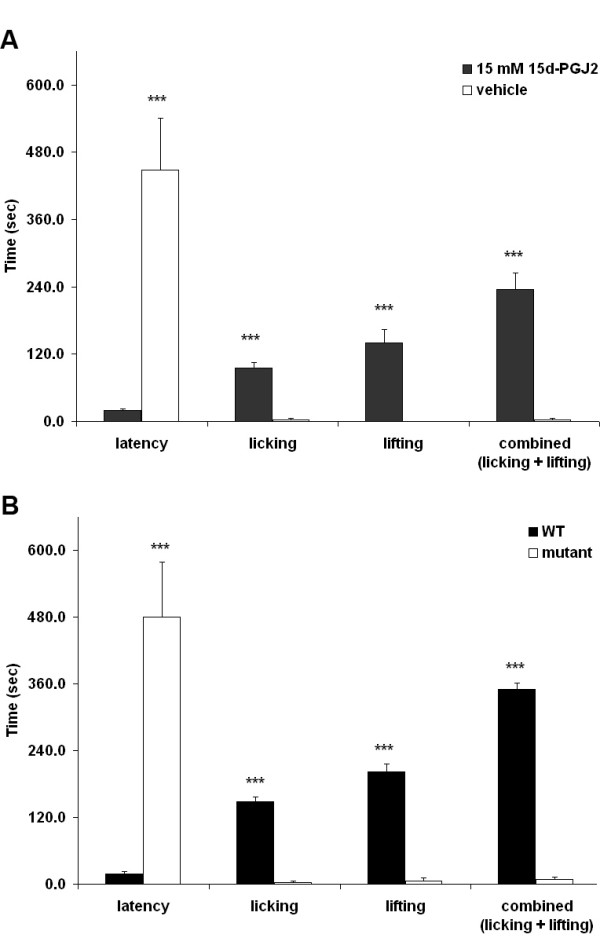
**Intraplantar injection of 15d-PGJ_2 _causes acute nociceptive responses via TRPA1**. (A) 10 μL of vehicle (10% DMSO in saline) or 15 nmol 15d-PGJ_2 _was injected into the hindpaw of C57BL/6J mice (n = 5 per group) and nociceptive behaviors (licking and lifting of the paw) for 10 minutes. 15d-PGJ_2 _caused significant nociceptive responses compared to vehicle. (B) 15d-PGJ_2_-induced nociceptive behaviors are absent in TRPA1 knockout mice (n = 5 per group). ***p < 0.001

The TRPA1 agonists including AITC, cinnamaldehyde and formaldehyde induce acute nociceptive behaviors including licking and flicking when injected in the hindpaw[4-6,15,16]. Peripheral nociceptive behaviors have not been reported in response to 15d-PGJ_2_. Therefore, we performed a dose-response study using 2.5–25 nmol 15d-PGJ_2 _hindpaw injection. A dose of 15 nmol was required in 10–12 week-old mice to elicit significant lifting and licking of the hindpaw compared to vehicle (Figure 5A). Therefore, all future studies utilizing wildtype and age-matched 10–12 week-old TRPA1 knockout littermates were performed using 15 nmol 15d-PGJ_2_. As shown in Figure 5, 15 nmol 15d-PGJ_2 _induced significant nociceptive behaviors including licking and lifting of the hindpaw compared to vehicle injection in C57BL/6J mice. The latency to the first response was scored as initial licking of the paw. Then the mice behaved by spending a significant amount of time both licking the injected paw and keeping it off the surface of the testing apparatus by lifting. To determine whether nociceptive behaviors were specific to TRPA1 activation, we performed similar experiments using wildtype and TRPA1 knockout littermates. As shown in Figure 5B, these behaviors were essentially absent in TRPA1-deficient mice, suggesting that acute peripheral nociception induced by 15d-PGJ_2 _occurs via specific activation of this channel.

## Discussion

Results of the present study reveal a previously uncharacterized role of 15d-PGJ_2 _in peripheral nociception. We show that 15d-PGJ2, similar to all known TRPA1 ligands, induces pain. We further demonstrate a causal link between 15d-PGJ_2_-induced nociception and TRPA1 activation at the cellular and behavioral levels.

Intriguingly, 15d-PGJ_2 _is best characterized as a potent anti-inflammatory agent and not as a molecule that induces acute or long-term inflammatory pain. Instead, the resolution of the inflammatory state appears to correlate with increasing levels of 15d-PGJ_2 _within tissue fluids[17]. Like other cyclopentane prostaglandins, 15d-PGJ_2 _does not function via a specific GPCR. Rather, the anti-inflammatory effects of 15d-PGJ_2 _are mediated by activation of the transcription factor peroxisome proliferator-activated receptor gamma (PPARγ) [18-20]. For example, 15d-PGJ_2 _represses the transcription of a number of pro-inflammatory factors including inducible nitric oxide synthase, cyclooxygenase-2 and tumor necrosis factor-α[21,22]. 15d-PGJ_2 _also acts independent of PPARγ to alter the activity of inflammatory molecules. It is thought to directly alkylate nucleophilic cysteine residues of NF-κB, thereby inhibiting DNA binding by NF-κB [23]. Similarly, TRPA1 is activated by the covalent binding of electrophiles to cysteines, providing a likely mechanism whereby 15d-PGJ_2 _activates this channel[9,10].

The results of our initial compound library screen were borne out by follow-up electrophysiological recordings showing that 15d-PGJ_2_directly activates TRPA1. In contrast to 15d-PGJ_2_, the J series PGD_2 _metabolites PGJ_2 _and 12d-PGJ_2_, which also contain reactive electrophilic carbons, failed to activate TRPA1 in our studies. A recent study by Taylor-Clark et al., which examined the activation of human TRPA1 using heterologous expression in HEK cells, found that 12d-PGJ_2 _was able to activate this channel[24]. Discrepancies between this study and our study could be due to concentrations tested (5 times greater concentrations were utilized by Taylor-Clark et al.), expression systems used or to subtle species differences in channel structure. The in vivo behavioral studies described here extend those of Taylor-Clark et al. by demonstrating that 15d-PGJ2 activation of TRPA1 is physiologically relevant. Lastly, during the preparation of this manuscript Andersson et al. published an article on the activation of TRPA1 by mediators of oxidative stress (including 15d-PGJ2) which confirm our findings presented here [26].

Consistent with a physiological role of 15d-PGJ_2 _in the activation of TRPA1, we identified a population of DRG neurons sensitive to 15d-PGJ_2_, AITC and capsaicin. TRPA1-expressing neurons occur as a subset of TRPV1-expressing neurons in the DRG; whereas TRPM8 is expressed in a separate population. These results support our electrophysiological studies showing that 15d-PGJ_2 _does not activate TRPV1 or TRPM8. In further support of specific activation of TRPA1, a negligible number of 15d-PGJ_2_/AITC responsive neurons were detected in cultures derived from TRPA1 knockout mice compared to wildtype (~2% vs. 90% respectively).

Specific agonists of TRPA1 cause acute nociceptive behaviors *in vivo *when administered cutaneously. Therefore, we investigated responses to intraplantar injection of 15d-PGJ_2_. Both C57BL/6J and TRPA1 wildtype mice responded robustly by licking of the injected paw as well as lifting of the paw from the surface of the testing apparatus. These behaviors were dramatically abolished in TRPA1 knockout mice, suggesting that 15d-PGJ_2_-induced acute peripheral nociception is mediated by TRPA1.

Previous studies have demonstrated a definitive role of TRPA1 in transmitting acute and inflammatory pain[4-8,15,16,25]. Here we show that 15d-PGJ_2_, a molecule with no known membrane receptor that is implicated in anti-inflammatory pathways, specifically activates TRPA1, an ion channel expressed in the cell membrane of nociceptive neurons. We also show that similar to other TRPA1 agonists, 15d-PGJ_2 _induces robust, acute nociceptive behaviors *in vivo*. Our data also support that 15d-PGJ_2_-induced peripheral nociception *in vivo *occurs through TRPA1 signaling. Collectively, our findings elaborate on a novel function of 15d-PGJ_2 _in peripheral nociception and identify TRPA1 as its principal receptor in pain-sensing DRG neurons.

## Competing interests

The authors declare they that have no competing interests.

## Authors' contributions

All authors read and approved the final manuscript. LCO and MCM performed all behavioral studies. EJC and DK performed electrophysiological studies. AD performed DRG analysis. RJH and TJY performed all PCR genotyping of knockout mice. GMS organized the project and identified 15d-PGJ2 in the initial library screen.
